# Integrating data science into the translational science research spectrum: A substance use disorder case study

**DOI:** 10.1017/cts.2020.521

**Published:** 2020-08-19

**Authors:** Emily Slade, Linda P. Dwoskin, Guo-Qiang Zhang, Jeffery C. Talbert, Jin Chen, Patricia R. Freeman, Kathleen M. Kantak, Emily R. Hankosky, Sajjad Fouladvand, Amy L. Meadows, Heather M. Bush

**Affiliations:** 1 Department of Biostatistics, University of Kentucky, Lexington, KY, USA; 2 Department of Pharmaceutical Sciences, University of Kentucky, Lexington, KY, USA; 3 University of Texas Health Science Center at Houston, Houston, TX, USA; 4 Institute for Pharmaceutical Outcomes and Policy, University of Kentucky, Lexington, KY, USA; 5 Institute for Biomedical Informatics, University of Kentucky, Lexington, KY, USA; 6 Department of Pharmacy Practice and Science, University of Kentucky, Lexington, KY, USA; 7 Department of Psychological and Brain Sciences, Boston University, Boston, MA, USA; 8 Department of Computer Science, University of Kentucky, Lexington, KY, USA; 9 Department of Psychiatry, University of Kentucky, Lexington, KY, USA; 10 Department of Pediatrics, University of Kentucky, Lexington, KY, USA

**Keywords:** Data science, team science, translational science research spectrum, substance use disorder, healthcare big data

## Abstract

The availability of large healthcare datasets offers the opportunity for researchers to navigate the traditional clinical and translational science research stages in a nonlinear manner. In particular, data scientists can harness the power of large healthcare datasets to bridge from preclinical discoveries (T0) directly to assessing population-level health impact (T4). A successful bridge from T0 to T4 does not bypass the other stages entirely; rather, effective team science makes a direct progression from T0 to T4 impactful by incorporating the perspectives of researchers from every stage of the clinical and translational science research spectrum. In this exemplar, we demonstrate how effective team science overcame challenges and, ultimately, ensured success when a diverse team of researchers worked together, using healthcare big data to test population-level substance use disorder (SUD) hypotheses generated from preclinical rodent studies. This project, called Advancing Substance use disorder Knowledge using Big Data (ASK Big Data), highlights the critical roles that data science expertise and effective team science play in quickly translating preclinical research into public health impact.

## Introduction

The clinical and translational science research spectrum outlines how scientific discoveries in the laboratory progress toward improved global health in distinct stages: basic scientific discovery (T0), translation to humans (T1), translation to patients (T2), translation to practice (T3), and translation to communities (T4) [1,2]. When the spectrum is navigated sequentially, discoveries in the preclinical stage, oftentimes in animal studies, generate hypotheses that are subsequently tested in small, well-controlled studies of humans [1]. These early human research studies aim to establish proof of concept and safety before investing substantial resources in large-scale studies [1]. When translating scientific research into communities, population-level impact in real-world scenarios is often measured using very large healthcare datasets, i.e., healthcare big data [1,3].

The translational science research spectrum does not have to be navigated linearly, though [4]. The availability and accessibility of healthcare big data offers an emerging opportunity to rethink the traditional approach to discovery [3]. As healthcare big data are now more accessible, investigators are frequently incorporating these data resources to investigate hypotheses generated in preclinical studies. When the study is ethically impractical or cost-prohibitive in clinical stages, this strategy has a distinct advantage. The data management and analytics tasks required for working with healthcare big data in T4 fall primarily to data scientists [3]. However, it is the successful, integrative collaboration between data scientists and researchers from every stage of the translational science research spectrum that ensures that the data-related tasks in T4 are comprehensive and performed in such a way to ensure that the end discovery is an impactful translation [5,6].

**Fig. 1.**
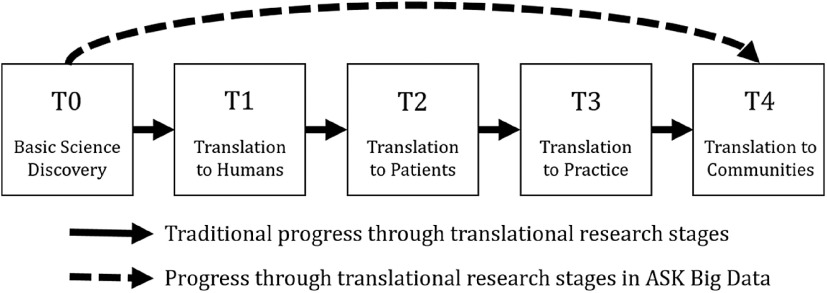
Clinical and translational science research spectrum. *Note*: The clinical and translational science research spectrum can be navigated sequentially (solid line). The Advancing Substance use disorder Knowledge using Big Data (ASK Big Data) project demonstrates one way in which these stages can be navigated in a nonlinear fashion (dashed line).

Here, we present the exemplar of a research study initiated at the University of Kentucky called Advancing Substance use disorder Knowledge with Big Data (ASK Big Data). The ASK Big Data project is a prime example of how big data may help shape the translational science research paradigm (T0 directly to T4), as shown in Fig. 1. The experiences of the ASK Big Data team highlight the importance of effective team science as a means to overcoming common challenges faced by data scientists on projects that circumvent the implementation of some stages of the translational science research spectrum. In this paper, we first provide a brief summary of the ASK Big Data team’s relevant research findings in order to give context to the discussion of lessons learned in the effort to bridge preclinical findings to big data discovery.

## Motivation

Attention-deficit/hyperactivity disorder (ADHD) is a prevalent neurodevelopmental disorder, estimated in 2015–2016 to affect 10.2% of children and adolescents aged 4–17 years old in the USA [7]. Estimated at only 6.1% in 1997–1998, the prevalence of ADHD in children and adolescents has increased significantly over the 20-year span both overall and in subgroups defined by age, sex, race/ethnicity, family income, and geographic region [7]. As the rate of ADHD diagnoses has increased, so has the rate of medication use for treating ADHD [8,9]. As of 2010, 74% of children and adolescents with ADHD were taking medication to treat the disorder [10].

Substance use disorder (SUD), resulting from recurrent, compulsive drug or alcohol use despite harmful consequence, is also a major public health concern in the USA, affecting approximately 20.3 million Americans aged 12 or older in 2018 [11]. SUD and ADHD are highly comorbid; adults with a history of ADHD are twice as likely to develop SUD compared to adults without ADHD [12,13]. There is some evidence that medication for ADHD contributes to the link between ADHD and SUD, but the biological mechanism by which this occurs is not well known [14–20]. Prior studies of ADHD medication initiation in children have found no evidence of increased risk [14–17] or even a decreased risk [18] for developing SUD later in life. In contrast, there is emerging evidence that when the initiation of ADHD medication occurs during adolescence, there is an increased risk of developing SUD [14,19]. Adolescence is a particularly important period for the development of brain structure and function, so it is possible that adolescents may be particularly sensitive to pharmacological changes during this time [20]. These diverging results motivated the desire to better understand how the timing of initiating treatment for ADHD during adolescence is linked to subsequent SUD in a well-controlled setting.

## Basic Scientific Discovery (T0)

To examine the underlying biological mechanisms and impact of treatment for ADHD in preclinical studies, experiments can be performed on rats that exhibit the ADHD phenotype (spontaneously hypertensive rats) and on rats that do not exhibit the ADHD phenotype (Wistar–Kyoto and Wistar rats) [21]. A number of rodent experiments were performed in the labs of the basic science researchers on our team to explore the effects of ADHD medication on the rats’ propensity to exhibit addiction-like behavior [22–27]. In one rodent study, rats exhibiting the ADHD phenotype were given methylphenidate, a popular stimulant medication used to treat ADHD in humans, starting at the beginning of their adolescent period [22]. After receiving methylphenidate treatment throughout adolescence, these rats acquired cocaine self-administration faster and showed greater motivation to self-administer cocaine as compared to rats receiving vehicle (no ADHD medication) and as compared to rats not exhibiting the ADHD phenotype that received methylphenidate [22].

Several subsequent rodent studies were performed to explore additional aspects of the relationship between ADHD medication initiation during adolescence and future susceptibility to cocaine addiction. Extending treatment into adulthood did not diminish the greater risk of cocaine self-administration in rats exhibiting the ADHD phenotype with adolescent-initiated methylphenidate [23]. However, the type of ADHD medication did appear to be an important factor, as vulnerability to cocaine addiction was not further elevated in these rats when treated during adolescence with atomoxetine or d-amphetamine [24–27]. Details of the experimental methodologies and results from these rodent studies are published elsewhere [22–27]. Altogether, these preclinical findings led to the basic scientists’ hypothesis that adolescence is a sensitive period for initiation of ADHD medication, which may increase the risk for SUD in adulthood.

## Translation to Humans, Patients, and Practice (T1–T3)

Translating our team’s preclinical findings into humans in a randomized controlled trial was not possible due to the ethical limitations of assigning the type and timing of ADHD medications. The time and costs required for conducting a randomized controlled trial presented further difficulties. Other groups have initiated observational studies to test similar hypotheses about ADHD medication and SUD in humans, with varied results. One study found evidence that the risk of adolescent SUD was higher among those who initiated ADHD medication during adolescence (vs. early childhood) and among those using only non-stimulant medications (vs. stimulant medications), but this study was limited by the cross-sectional nature of data collection [28]. Another study did use temporal ADHD treatment records from large healthcare claims data, and it found evidence that ADHD medication was associated with lower concurrent risk of SUD [29]. However, this study included a heterogeneous population of adolescents and adults (aged 13–64 years) and did not specifically explore the initiation and patterns of ADHD medication prescribing among adolescents [29].

A more focused study using temporal data was needed to specifically address the hypotheses generated in preclinical studies regarding ADHD medication timing in adolescence and subsequent risk of SUD. The basic scientists responsible for the aforementioned rodent studies at the University of Kentucky and Boston University wanted to be able to address potential latent factors encoded in complex medication use patterns that can only be studied using temporal healthcare big data. They knew that it was necessary to collaborate with data scientists to accomplish this goal of jumping directly to T4, and through this undertaking, the ASK Big Data team formed (Table 1).

**Table 1.** Translational data science team for the ASK Big Data project

Investigator	Expertise	Center for Clinical and Translational Science Role
Biomedical informatician (MPI)	Ontology and terminology systems; T4	Director, Biomedical Informatics
Biomedical informatician, (MPI)	Population (SUD) outcomes and policy; T4	Director, Biomedical Informatics
Neuropharmacologist, (MPI)	SUD research, drug discovery; T0–T1	Director, Drug Discovery and Development
Behavioral neuroscientist	SUD research, drug discovery; T0	
Biomedical informatician	Natural language processing, computational data science; T4	
Biostatistician	Population data, secondary data analysis and design; T0–T4	Co-Director, BERD
Biostatistician	Population data, causal inference; T0–T4	BERD
Clinical pharmacist	SUD research, pharmacy practice; T2–T4	
Clinician	Pediatric psychiatry, ADHD, SUD research; T2–T4	

NIH National Center for Advancing Translational Sciences (NCATS) and the Clinical and Translational Science Award (CTSA) programs are particularly good environments for connecting researchers across the translational science research spectrum [30]. The role of the Center for Clinical and Translational Science at the University of Kentucky is evident in the ASK Big Data team.

ASK Big Data, advancing substance use disorder knowledge with big data; MPI, multiple principal investigator; SUD, substance use disorder; BERD, biostatistics, epidemiology, and research design core; ADHD, attention-deficit/hyperactivity disorder.

## Translation to Population Health (T4)

To investigate the link between timing of ADHD medication in adolescence and subsequent risk of SUD on a population level, the ASK Big Data team utilized the IBM (formerly Truven Health Analytics) MarketScan Commercial Claims and Encounters database (referred to hereafter as IBM MarketScan) [31]. The IBM MarketScan database includes inpatient and outpatient medical encounter data as well as prescription drug information from Americans with employer-sponsored private health insurance and their dependents. Using the International Classification of Disease (ICD-9) diagnosis codes, the ASK Big Data team extracted all ADHD-related records from the IBM MarketScan database between January 2009 and December 2015 [31]. In total, 11,778,912 records were extracted, including information from 118,063 enrollees with adolescent (13–20 years) initiation of ADHD medication [31]. In addition to temporal data from longitudinal ADHD medication records, the extracted data also included stationary features such as sex, ADHD initiation age, and medication type [31]. Among the 118,063 enrollees with adolescent initiation of ADHD medication, 9376 (7.9%) were classified as ADHD-SUD positive, defined as being diagnosed with SUD for the first time after having an ADHD medication prescription for at least 5 months [31].

To predict SUD using the temporal ADHD medication records, the ASK Big Data team utilized a long short-term memory (LSTM) model which is a type of recurrent neural network that accommodates longitudinal data [31]. Further details regarding specification of the LSTM models can be found in Fouladvand *et al.* [31]. Using the adolescent temporal ADHD medication records from IBM MarketScan, the LSTM model was able to predict SUD with an accuracy of 0.84 (F1 score = 0.82 ± 0.01) [31]. The high performance of the LSTM model relative to traditional classification models (random forest accuracy = 0.60, support vector machine accuracy = 0.56) indicates that there may be critical latent factors in the detailed temporal data that capture the relationship between ADHD medication prescribing and subsequent SUD in adolescents [31]. In addition to the temporal data, the ASK Big Data team also explored whether stationary features such as age, sex, and medication types in the IBM MarketScan data aided in the detection of SUD among adolescents being treated for ADHD [31]. To summarize the results of Fouladvand *et al.*, temporal features of ADHD medication prescribing such as gaps and duration of medication, rather than stationary features such as demographics, were the important factors in predicting the development of SUD among adolescent initiators of ADHD medication [31].

Overall, these findings supported population-level investigation of the hypothesis generated in T0 that the adolescent period is a sensitive time for initiation of ADHD medication as it relates to development of SUD. Moreover, these data discoveries have highlighted the need to iteratively identify and refine clinically-meaningful SUD phenotypes with domain expertise. Considering the trajectory of experiences leading to SUD with population-level data continues to be an active research program for the ASK Big Data team. With knowledge from preclinical findings and data-driven discoveries, our approach continues to provide novel opportunities to investigate relationships between life course exposures and SUD. The ASK Big Data team’s approach leveraged big population-level healthcare claims data to confirm that temporal ADHD medication prescribing records can be used to predict subsequent SUD in adulthood. The findings from these initial outcomes continue to generate new avenues for research and continue to offer this team an innovative strategy for exploring drivers (precursors and exposures) of SUD experience.

## Lessons Learned

The ASK Big Data team first started taking shape as one of the key investigators on the ADHD rodent studies, a basic scientist with a background in pharmacology, wanted to see if and how findings from animal studies might translate to humans. The features of these experiments would be unethical to conduct in humans, and the time period to observe similar life stages in humans would be cost-prohibitive. The pharmacologist teamed up with two biomedical informaticians, one with expertise in health policy and one with expertise in computational data science. Their primary objective was to determine if patient experiences encoded in big data would provide human health evidence for these preclinical trends. This established the T0 and T4 pillars of the ASK Big Data team, with researchers from both ends of the translational science research spectrum serving as principal investigators on the project. Rather than having a single principal investigator, this multiple principal investigator approach ensured that no one side (domain leader or data scientist) felt stronger ownership over the project than another. However, even with expertise on both ends of the translational science research spectrum, jumping from T0 to T4 is not a trivial task. In fact, this exemplar offers insight into four key attributes of an effective team when attempting to bridge and integrate elements of the translational research spectrum.

### Lesson 1: Recognize that everyone is an expert and a novice

While research teams are often constructed based on expertise, the ASK Big Data team was comprised of investigators of similar rank, holding leadership positions, with experience as principal investigators. As such, each person was recognized as an expert in their field but had gaps in knowledge related to this research project. For example, preclinical scientists were less familiar with population-level data, SUD (clinical or preclinical) research was not a domain area of expertise for all, and data science methodology was not a standard analytic approach for secondary data analysis. The contrast of high levels of expertise in one area with relative naiveté in another resulted in an equalizing effect. All researchers capably contributed as experts but benefited in learning from each other. Further, this reliance on each other for information created a foundation of trust throughout the team. A hallmark of team meetings was to remain on a topic until everyone had an understanding; no researcher was left behind, and no question was unwelcome. While this was not always expeditious, the efforts to impress or demonstrate superior roles that sometimes occur in teams did not arise.

### Lesson 2: Avoid the divide-and-conquer approach

Often teams will divide the work that needs to be completed for efficiency. For example, in an effort to save a biostatistician’s time, an investigator may provide parts of a proposal and ask for the methods section to be written. Many teams work within product or function structures where like groups work together on similar parts [32]. This style of team organization requires the principal investigator to act as coordinator or lead to converge the different components. In scientific research, this division of effort often occurs to complete sections of a manuscript or components of a proposal; pieces created individually are then circulated among all. The ASK Big Data team used a less common approach of a flat organizational structure, where investigators work at the same level and as a collective. One of the unique features of the ASK Big Data team was to meet together and write or work through problems as a group, i.e., “group write”. This type of integrated work style contributed to shared ownership of research products, made possible by the open exchange of ideas and concepts (see Lesson 1). Data scientists were not relegated only to “their” sections, and preclinical investigators did not shirk away for the methodological details. This unique approach circumvented typical clashes between personalities because efforts were so intertwined.

A critical factor for success on the ASK Big Data team was that every member of the team was deeply invested in the project. None of the team members acted as consultants; rather, the basic scientists, clinicians, pharmacists, biostatisticians, informaticians, and computational data scientists were all at the table to contribute their unique perspective. It quickly became obvious how important this personal investment was for the ASK Big Data team. Even when just one team member was absent from a meeting, it was not uncommon for the team to head in a direction that later had to be rerouted when the full team next assembled. While this provides a feature for successful team science that can create an exciting learning experience, it does require a willingness and ability to stretch into other disciplines. A biostatistician who does not embrace the domain area or the investigator who considers methodology as technical details cannot successfully engage in this type of structure.

### Lesson 3: Embrace discipline-specific approaches to science

Although there was significant exchange and shared learning in the ASK Big Data team, the span across the translational science spectrum identified stark differences in how T0 and T4 researchers view the process of scientific inquiry. Basic scientists who primarily work in the T0 stage are accustomed to testing very specific hypotheses with scientifically-rigorous, well-controlled studies. On the other hand, computational data scientists who spend most of their time in the T4 stage are comfortable using complex methods to extract information from messy data that are often collected for a primary purpose not related to research. The existence of these very different but both valuable philosophies for conducting scientific research can lead to discord between the approaches of basic science and computational data science. Biostatistics played a key role in bridging this gap to avoid discord on the ASK Big Data team. Since biostatisticians typically have some experience with both approaches to scientific research, they are uniquely suited to facilitate interactions between basic scientists and computational data scientists. For example, data discoveries in the ASK Big Data project were improved by specifically defining risk sets and control groups; appropriately framing the temporal sequence of events within health records increased the validity of the findings. With data as the connector, biostatistics provided a common framework for discussing analytic plans and strategizing about how to utilize, interpret, and present the results.

### Lesson 4: Frame data discovery in a clinical context

Translation of T0–T4 requires the population-level data science in T4 to be performed in a clinically-appropriate and meaningful way. In other words, T1, T2, and T3 are relevant and necessary even if the research process does not formally incorporate these stages. Incorporating perspectives from experts across the translational research spectrum was paramount to overcoming this challenge on the ASK Big Data project. For example, the enrollee medication record matrix in the IBM MarketScan data consisted of 0/1 values where rows are visits and columns are medications [31]. This poses a challenge for data scientists because these types of matrices can be quite sparse, and a high degree of data sparsity can lower the performance of a deep learning model [33]. Key members of the ASK Big Data team provided clinical expertise to help overcome this challenge. For example, the clinician was able to shed light on how diagnosis codes are entered, which informed the team’s definition of the SUD outcome in the IBM MarketScan data. The pharmacist provided practical information on how ADHD medication is prescribed, such as the common 3-month gap in medication observed in many adolescents due to summer break in the school year. Taking these perspectives into account, the ASK Big Data team’s data scientists were able to reduce data sparsity by removing four types of records: (1) all empty records before the first ADHD medication prescription, (2) all empty records after the last ADHD medication prescription, (3) sequences in which the enrollee used ADHD medication for less than 5 months, and (4) enrollees who started using ADHD medication less than 5 months before being diagnosed with SUD [31]. While the data sparsity issue was identified by the team’s computational data scientists, developing an appropriate solution required input from other ASK Big Data team members who had intimate knowledge of ADHD medication prescribing in practice. Engaging researchers from all stages of the translational science research spectrum embedded T1–T3 principles and expertise within the process and expanded the domain expertise of the data scientists on the ASK Big Data team, offering the perspective needed to implement an appropriate analysis.

## Conclusion

Effective team science requires a diversity of thought and true integration across disciplines [5,6]. The ASK Big Data team demonstrated this not only by recruiting a highly interdisciplinary team of researchers representing every stage of the translational science research spectrum, but also by ensuring that each of these team members had an equally important seat at the table, with everyone at the table equally engaged.

Navigating the translational science research spectrum sequentially can be time-consuming, expensive, and potentially unethical, and translating preclinical discoveries into humans is particularly prone to failure [34]. Creating a pathway directly from preclinical work to analyzing population-level impact circumvents these challenges. Data scientists should feel empowered by the accessibility of healthcare big data in making this pathway viable. When navigating this streamlined approach, impactful research in T4 cannot be made by data scientists alone. Working effectively with researchers from every stage of the clinical and translational science research spectrum ensures that data scientists ask questions and design analyses and data discovery strategies that are appropriate in the context of clinical practice.

This case study should not be viewed as a call for abandonment of the traditional progress through clinical and translational science research stages; each of these stages is certainly necessary for processes like drug development. Instead, we use the ASK Big Data project to demonstrate one of many ways that data science alters and augments the paradigm. In this example, we present an alternative pathway, but data science can also serve to make these pathways bidirectional. We fully expect that the SUD research community can benefit from testing data-discovered hypotheses within preclinical or clinical studies. Our experience demonstrates how team science and, consequently, translational data science, can overcome common challenges for investigators as T0–T4 expertise is integrated to include preclinical, clinical, informatics, and biostatistics to solve complex, global human health problems.

## References

[1] Translational Science Spectrum. National Center for Advancing Translational Sciences [Internet], 2020 [cited Feb 16, 2020]. (http://ncats.nih.gov/translation/spectrum)

[2] Clinical and Translational Research Spectrum. Harvard Catalyst [Internet], 2020 [cited Feb 16, 2020]. (http://catalyst.harvard.edu/pathfinder/)

[3] Raghupathi W , Raghupathi V . Big data analytics in healthcare: promise and potential. Health Information Science and Systems 2014; 2(3): 1–10. 25825667 10.1186/2047-2501-2-3 PMC4341817

[4] Milat AJ , Li B . Narrative review of frameworks for translating research evidence into policy and practice. Public Health Research and Practice 2017; 27(1): e2711704. 10.17061/phrp2711704 28243670

[5] Disis ML , Slattery JT . The road we must take: multidisciplinary team science. Science Translational Medicine 2010; 2(22): 22cm9. 10.1126/scitranslmed.3000421 20374998

[6] Stokols D , Hall KL , Taylor BK , Moser RP . The science of team science: overview of the field and introduction to the supplement. American Journal of Preventive Medicine 2008; 35(2): S77–S89. 18619407 10.1016/j.amepre.2008.05.002

[7] Xu G , et al. Twenty-year trends in diagnosed attention-deficit/hyperactivity disorder among US children and adolescents, 1997-2016. JAMA Network Open 2018; 1(4): e181471. 30646132 10.1001/jamanetworkopen.2018.1471 PMC6324288

[8] Centers for Disease Control and Prevention. Increasing prevalence of parent-reported attention-deficit/hyperactivity disorder among children – United States, 2003 and 2007. Morbidity and Mortality Weekly Report 2010; 59(44): 1439–1443. 21063274

[9] Zuvekas SH , Vitiello B . Stimulant medication use in children: a 12-year perspective. American Journal of Psychiatry 2012; 169(2): 160–166. 10.1176/appi.ajp.2011.11030387 PMC3548321 22420039

[10] Visser SN , et al. Treatment of attention-deficit/hyperactivity disorder among children with special health care needs. The Journal of Pediatrics 2015; 166(6): 1423–1430. 25841538 10.1016/j.jpeds.2015.02.018 PMC4469986

[11] Substance Abuse and Mental Health Services Administration. Key Substance use and Mental Health Indicators in the United States: Results from the 2018 National Survey on Drug Use and Health (HHS Publication No. PEP19-5068, NSDUH Series H-54). Rockville, MD: Center for Behavioral Health Statistics and Quality, Substance Abuse and Mental Health Services Administration. Retrieved from https://www.samhsa.gov/data/.

[12] Biederman J , et al. Does attention-deficit hyperactivity disorder impact the developmental course of drug and alcohol abuse and dependence? Biological Psychiatry 1998; 44: 269–273. 9715358 10.1016/s0006-3223(97)00406-x

[13] Lee SS , et al. Prospective association of childhood attention-deficit/hyperactivity disorder (ADHD) and substance use and abuse/dependence: a meta-analytic review. Clinical Psychology Review 2011; 31: 328–341. 21382538 10.1016/j.cpr.2011.01.006 PMC3180912

[14] Mannuzza S , et al. Age of methylphenidate treatment initiation in children with ADHD and later substance abuse: prospective follow-up into adulthood. American Journal of Psychiatry 2008; 165: 604–609. 10.1176/appi.ajp.2008.07091465 PMC2967384 18381904

[15] Humphreys KL , Eng T , Lee SS . Stimulant medication and substance use outcomes. Journal of the American Medical Association 2013; 70: 740–749. 10.1001/jamapsychiatry.2013.1273 PMC6688478 23754458

[16] Molina BSG , et al. Adolescent substance use in the multimodal treatment study of attention-deficit/hyperactivity disorder (ADHD) (MTA) as a function of childhood ADHD, random assignment to childhood treatments, and subsequent medication. Journal of the American Academy of Child and Adolescent Psychiatry 2013; 52: 250–263. 23452682 10.1016/j.jaac.2012.12.014 PMC3589108

[17] Volkow ND , Swanson JM . Does childhood treatment of ADHD with stimulant medication affect substance abuse in adulthood? American Journal of Psychiatry 2008; 165: 553–555. 10.1176/appi.ajp.2008.08020237 PMC2667111 18450933

[18] Wilens TE , et al. Does stimulant therapy of attention-deficit/hyperactivity disorder beget later substance abuse? A meta-analytic review of the literature. Pediatrics 2003; 111: 179–185. 12509574 10.1542/peds.111.1.179

[19] Kollins SH . A qualitative review of issues arising in the use of psycho-stimulant medications in patients with ADHD and co-morbid substance use disorders. Current Medical Research and Opinion 2008; 24: 1345–1357. 18384709 10.1185/030079908x280707

[20] Andersen SL . Trajectories of brain development: point of vulnerability or window of opportunity? Neuroscience & Biobehavioral Reviews 2003; 27: 3–18. 12732219 10.1016/s0149-7634(03)00005-8

[21] Sagvolden T , et al. The spontaneously hypertensive rat (SHR) as an animal model of childhood hyperactivity (ADHD): changed reactivity to reinforcers and to psychomotor stimulants. Behavioral and Neural Biology 1992; 58: 103–112. 1360797 10.1016/0163-1047(92)90315-u

[22] Harvey RC , et al. Methylphenidate treatment in adolescent rats with an attention deficit/hyperactivity disorder phenotype: cocaine addiction vulnerability and dopamine transporter function. Neuropsychopharmacology 2011, 36: 837–847. 21150910 10.1038/npp.2010.223 PMC3055734

[23] Baskin BM , Dwoskin LP , Kantak KM . Methylphenidate treatment beyond adolescence maintains increased self-administration in the Spontaneously Hypertensive Rat model of Attention Deficit/Hyperactivity Disorder. Pharmacology Biochemistry and Behavior 2015; 131: 51–56. 10.1016/j.pbb.2015.01.019 PMC4369437 25643872

[24] Somkuwar SS , et al. Adolescent atomoxetine treatment in a rodent model of ADHD: effects on cocaine self-administration and dopamine transporters in frontostriatal regions. Neuropsychopharmacology 2013; 38: 2588–2597. 23822950 10.1038/npp.2013.163 PMC3828528

[25] Jordan CJ , et al. Cocaine-seeking behavior in a genetic model of attention-deficit/hyperactivity disorder following adolescent methylphenidate or atomoxetine treatments. Drug and Alcohol Dependence 2014; 140: 25–32. 24811203 10.1016/j.drugalcdep.2014.04.020 PMC4075321

[26] Jordan CJ , et al. Adolescent d-amphetamine treatment in a rodent model of attention deficit/hyperactivity disorder: impact on cocaine abuse vulnerability in adulthood. Psychopharmacology 2016; 233: 3891–3903. 27600990 10.1007/s00213-016-4419-2 PMC5026317

[27] Jordan CJ , et al. Adolescent d-amphetamine treatment in a rodent model of ADHD: pro-cognitive effects in adolescence without an impact on cocaine cue reactivity in adulthood. Behavioural Brain Research 2016; 297: 165–179. 26467602 10.1016/j.bbr.2015.10.017 PMC4679481

[28] McCabe SE , et al. Age of onset, duration, and type of medication therapy for attention-deficit/hyperactivity disorder (ADHD) and substance use during adolescence: a multi-cohort national studies. Journal of the American Academy of Child and Adolescent Psychiatry 2016; 55(6): 479–486. 27238066 10.1016/j.jaac.2016.03.011 PMC4921895

[29] Quinn PD , et al. ADHD medication and substance-related problems. American Journal of Psychiatry 2017; 174(9): 877–885. 10.1176/appi.ajp.2017.16060686 PMC5581231 28659039

[30] Zerhouni EA . Translational and clinical science – time for a new vision. New England Journal of Medicine 2005; 353: 1621–1623. 10.1056/NEJMsb053723 16221788

[31] Fouladvand S , et al. Predicting substance use disorder using long-term attention deficit hyperactivity disorder medication records in Truven. Health Informatics Journal 2019; 26(2): 787–802. 31106686 10.1177/1460458219844075 PMC6861600

[32] Walker AH , Lorsch JW . Organizational choice: product versus function (1968). In: Shafritz JM , Ott JS , Jang YS , eds. Classics of Organizational Theory. Boston, MA: Cengage; 2016. 178–188.

[33] Miotto R , et al. Deep learning for healthcare: review, opportunities and challenges. Briefings in Bioinformatics 2017; 19: 1236–1246. 10.1093/bib/bbx044 PMC6455466 28481991

[34] Contopoulos-Ioannidis DG , et al. Life cycle of translational research for medical interventions. Science 2008; 321: 1298–1299. 18772421 10.1126/science.1160622

